# Assessment of left ventricular hemodynamic forces in healthy subjects and patients with dilated cardiomyopathy using 4D flow MRI

**DOI:** 10.14814/phy2.12685

**Published:** 2016-02-01

**Authors:** Jonatan Eriksson, Ann F. Bolger, Tino Ebbers, Carl‐Johan Carlhäll

**Affiliations:** ^1^ Divsion of Cardiovascular MedicineDepartment of Medical and Health SciencesLinköping UniversityLinköpingSweden; ^2^ Center for Medical Image Science and Visualization (CMIV)Linköping UniversityLinköpingSweden; ^3^ Department of MedicineUniversity of CaliforniaSan FranciscoCalifornia; ^4^ Division of Media and Information TechnologyDepartment of Science and Technology/Swedish e‐Science Research Centre (SeRC)Linköping UniversityLinköpingSweden; ^5^ Department of Clinical PhysiologyDepartment of Medical and Health SciencesLinköping UniversityLinköpingSweden

**Keywords:** 4D flow MRI, cardiac function, cardiac remodeling, dilated cardiomyopathy, hemodynamic forces

## Abstract

We hypothesized that the direction of global left ventricular (LV) hemodynamic forces during diastolic filling are concordant with the main flow axes in normal LVs, but that this pattern would be altered in dilated and dysfunctional LVs. Therefore, we aimed to assess the LV hemodynamic filling forces in a group of healthy subjects and compare them to the results from a group of patients with dilated cardiomyopathy (DCM). Ten healthy subjects and 10 DCM patients were enrolled. Morphological short‐ (SAx) and long‐axis (LAx) images and 4D flow MRI data were acquired at 1.5T. The LV pressure gradients were computed from the 4D flow data using the Navier–Stokes equations. By integrating the pressure gradients over the LV volume at each time frame, the magnitude and direction of the global hemodynamic force was calculated over the cardiac cycle. The hemodynamic forces acting in the SAx‐ and LAx‐directions were used to calculate the “SAx‐max/LAx‐max”‐ratio for the early (E‐wave) and late (A‐wave) diastolic filling. In the LAx‐plane, the temporal progression of the hemodynamic force followed a consistent pattern in the healthy subjects. The “SAx‐max/LAx‐max”‐ratio was significantly larger at both E‐wave (0.53 ± 0.15 vs. 0.23 ± 0.12, *P* < 0.0001) and A‐wave (0.44 ± 0.21 vs. 0.26 ± 0.09, *P* < 0.03) in the DCM patients compared to the healthy subjects. 4D flow MRI data allow quantification of LV hemodynamic forces acting on the LV myocardial wall. The LV hemodynamic filling forces showed a similar temporal progression among healthy subjects, whereas DCM patients had forces that were more heterogeneous in their direction and magnitude during diastole.

## Introduction

Heart failure represents the final stage of the continuum of cardiovascular diseases. Cardiac remodeling is a key component of heart failure that progresses from adaptive to maladaptive as the disorder worsens (Hill and Olson 2008). Increased myocardial wall stress during diastole contributes to the development and progression of adverse cardiac remodeling (Mann 2004).

During LV diastolic filling, blood in the left atrium (LA) is accelerated due to the pressure difference between the LA and the LV. After entering the LV, the speed is reduced. During this deceleration (i.e., negative acceleration), the LV myocardium exerts a force to decelerate the blood, while, following Newton′s third law (the action‐reaction law), a hemodynamic force, with equal magnitude but opposite direction, is exerted by the blood on the surrounding LV myocardial walls. During LV systole, the blood is accelerated toward the LV outflow tract by the force the contracting myocardium exerts on the blood, while the blood exerts a hemodynamic force on the surrounding LV myocardium.

As the hemodynamic forces are related to blood acceleration and deceleration in the LV, one may expect that during LV filling and emptying these forces would mostly be directed along the “base‐to‐apex axis”, concordant with the predominant flow directions in the normal LV (Eriksson et al. 2014). Altered flow patterns and loading conditions can be demonstrated in dilated and dysfunctional LVs. Thus, in hearts with pathological LV configuration and function, the LV hemodynamic forces that result from the altered intraventricular flow and loading may be abnormal in direction, temporal progression, and magnitude, possibly contributing to increased diastolic wall stress (Grossman et al. 1975). Until now, assessment of the global LV hemodynamic force has been based on theoretical considerations (Rushmer et al. 1964) or the direction of the force estimated in 2D planes (Pedrizzetti et al. 2014, 2015).

Time‐resolved, three‐directional velocity data in a three‐dimensional volume (4D flow) acquired by MRI enables assessment of multidimensional blood flow dynamics in the heart and great vessels (Firmin et al. 1993; Wigström et al. 1999; Frydrychowicz et al. 2007). This approach has been utilized to study intracardiac blood flow in healthy subjects and various patient groups (Bolger et al. 2007; Carlhäll and Bolger 2010; Ebbers 2011; Fredriksson et al. 2011; Arvidsson et al. 2013; Eriksson et al. 2013). Intracardiac absolute pressure is a clinically important hemodynamic marker, but it requires invasive measurement. Noninvasively measured velocity data and governing equations can be used to compute pressure gradients and relative pressure fields (Tyszka et al. 2000; Ebbers et al. 2001; Buyens et al. 2005). Relative pressure fields have been shown to be helpful in understanding regional flow phenomena in the LV (Eriksson et al. 2014). A more global parameter, such as the LV hemodynamic force (Pedrizzetti et al. 2015), may add to the understanding of the interacting forces between the LV blood flow and myocardium. The LV hemodynamic force is computed by integrating the pressure gradients over the entire LV volume at each time frame, and represents the force that the accelerating and decelerating blood in the ventricle exerts on the surrounding myocardial walls.

This study aims to extend the previously proposed hypothesis that the direction of global LV hemodynamic forces are concordant with the main flow axes in normal LVs, but that this pattern would be altered in dilated and dysfunctional LVs. Therefore, we sought to assess the global hemodynamic force during diastolic filling using three‐dimensional and three‐directional measurements of the moving blood in LVs of healthy subjects, and to compare those findings to results in a spectrum of patients with dilated cardiomyopathy.

## Methods

### Study population

Ten healthy subjects and 10 patients with idiopathic dilated cardiomyopathy (DCM) with a spectrum of LV remodeling and dysfunction were enrolled in the study. Clinical and demographical parameters are presented in Table 1. Inclusion criteria for (1) healthy subjects: normal electrocardiographic and echocardiographic examinations; (2) DCM patients: ≤65 years of age. At the time of diagnosis, idiopathic DCM was defined as the presence of symptoms and signs of heart failure (HF) in the presence of echocardiographic findings of LV enlargement and systolic dysfunction, and in the absence of clinical or echocardiographic evidence of ischemic, valvular or hypertensive cardiomyopathy. Exclusion criteria for (1) all subjects: contraindication to MRI examination; (2) healthy subjects: a history of prior heart disease, current heart disease or current use of cardiac medication; and (3) DCM patients: Significantly irregular cardiac rhythm, a history of myocardial infarction, ≥ moderate arterial hypertension, as well as ≥ moderate valvular disorder, < mild LV systolic dysfunction (LVEF >50%) and < mild LV dilatation defined by echocardiography. The grading of any valvular disease was performed according to echocardiographic guidelines by an experienced echocardiographer. Prior myocardial infarction was excluded based on history of ischemic heart disease, electrocardiography and wall motion by echocardiography at rest. The regional ethical review board approved the study. All subjects gave written informed consent before participation in the study. Our group pursues continuous development of sophisticated MRI based methodology, and some of the patient datasets have been used with different analysis tools in previous studies; three of the patient datasets were analyzed and reported in (Eriksson et al. 2010), 10 of the patient datasets were used in (Eriksson et al. 2013) and nine of the patient datasets were used in (Zajac et al. 2014).

**Table 1:** Demographical and clinical data for the healthy subjects and the DCM patients

	Healthy *n* = 10	DCM *n* = 10	*P*‐value
Age (years)	48 ± 15	49 ± 14	0.98
Gender (female:male)	4:6	6:4	N/A
Weight (kg)	74 ± 7	82 ± 18	0.21
Height (m)	175 ± 9	172 ± 7	0.45
Heart Rate (bpm)	67 ± 10	61 ± 11	0.22
Systolic BP (mmHg)	126 ± 9	122 ± 14	0.51
Diastolic BP (mmHg)	78 ± 8	77 ± 9	0.89
LVEF (%)	61 ± 3	41 ± 5	0.000
LVEDV (mL)	137 ± 15	177 ± 33	0.003
LV sphericity index	0.56 ± 0.06	0.75 ± 0.12	0.0003

bpm, beats per minute.

### Data acquisition

To confirm that each subject fulfilled the inclusion criteria, they all underwent a clinical electrocardiographic and echocardiographic examination. The echocardiographic examination was conducted using a Vivid 7 scanner and a 2.0 MHz probe (GE, Vingmed Ultrasound, Horten, Norway).

All subjects underwent a cardiac MRI examination on a clinical 1.5 T scanner (Philips Achieva, Philips Healthcare, Best, the Netherlands), where a five element cardiac SENSE coil was used as receiver and the body coil as transmitter. Morphological short‐ and long‐axis (SAx and LAx, respectively) balanced steady‐state‐free precession images as well as time‐resolved, three‐directional, three‐dimensional velocity (4D flow) data were acquired.

Morphological data were acquired in standard LAx two‐, three‐, and four‐chamber views as well as two stacks of SAx‐images. One SAx stack was acquired before the 4D flow scan and one after, in order to provide a backup should the first SAx stack show severe mismatch to the 4D flow data. All morphological images were acquired in 30 time frames and with 8 mm slice thickness. There was no slice gap between the short‐axis images in the stack. The LAx‐images were acquired with spatial resolution of 2.19 × 1.78 mm^2^ and the SAx‐stack with 1.67 × 1.78 mm^2^. The images were reconstructed into 1.25 × 1.25 mm^2^. All morphological images were acquired with retrospective cardiac gating. The 4D flow data were acquired during free‐breathing, using a navigator‐gated, gradient‐echo pulse sequence with interleaved three‐directional flow‐encoding and retrospective, vector‐cardiogram controlled cardiac gating. General acquisition parameters: VENC, 100 cm/s; echo time (TE), 3.7 ms; repetition time (TR), 6.3 ms; flip angle, 8°; SENSE factor, 2; k‐space segmentation factor, 2; and spatial resolution (acquired and reconstructed), 3.0 × 3.0 × 3.0 mm^3^. These settings gave a temporal resolution of 50.4 ms. The mean acquisition time for a 4D flow dataset was 31 ± 8 (median: 30 range: 16–57) minutes including the navigator efficiency.

After acquisition, the 4D flow data were reconstructed into 40 time frames on the MRI system. After reconstruction, the data were corrected for concomitant gradient field effects on the MRI system and exported to an offline station and postprocessed by the use of in‐house developed software written in Matlab (The Mathworks Inc., Natick, MA). In the postprocessing step, data were corrected for phase wraps (Xiang 1995) and background phase errors by the use of a least‐square fit of a 4th order polynomial to the static tissue in the thorax by using a weighted soft mask (Ebbers et al. 2008).

### Data analysis

**Figure 1:**
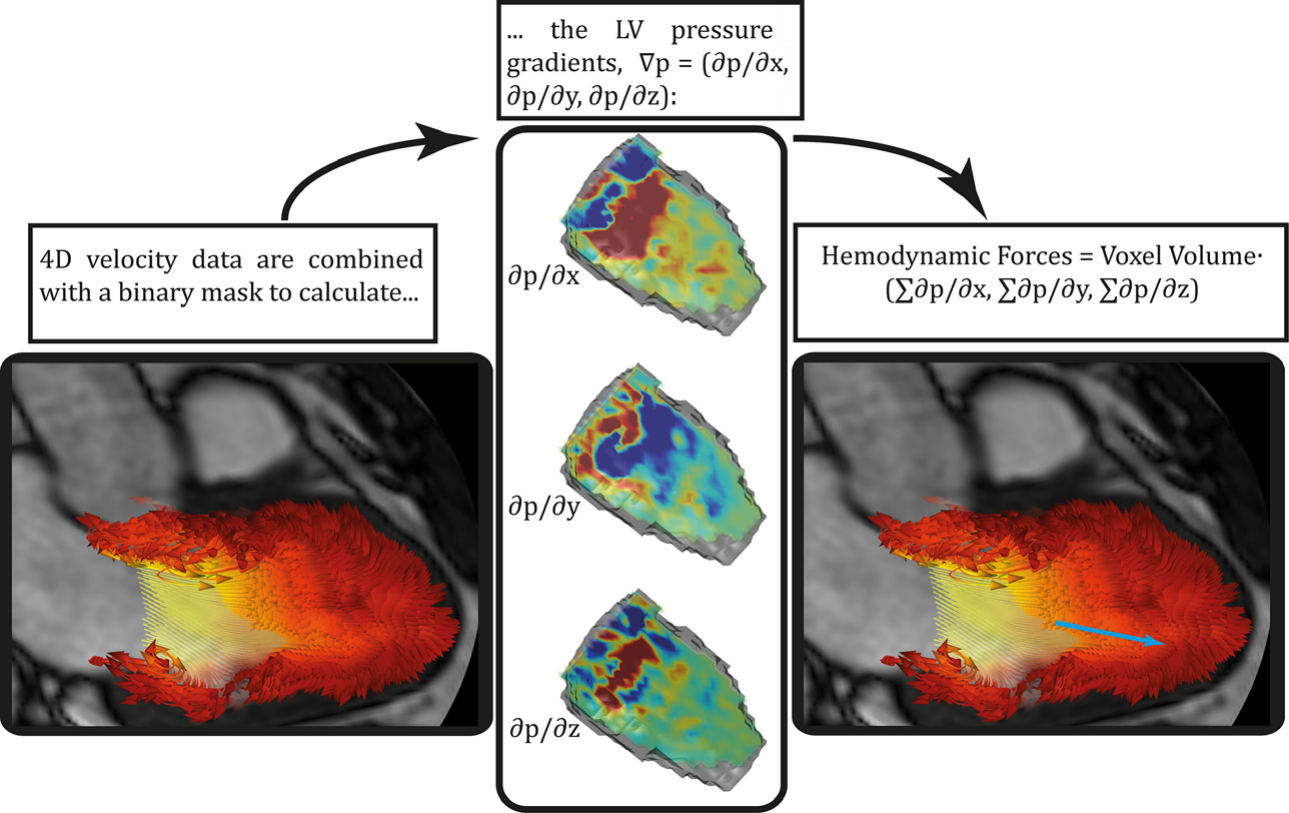
Flow chart describing the data analysis. After reconstruction and postprocessing of the 4D flow data, the LV was segmented over the cardiac cycle from the SAx‐images. The segmentation is converted to a binary mask and resampled to fit the 4D flow data volume. From the 4D flow data and the binary mask, the pressure gradients (∇p = ∂p/∂x, ∂p/∂y, ∂p/∂z) are calculated. The hemodynamic force (blue arrow), that is, the force that the moving blood is exerting on its surroundings, is calculated by integration of the pressure gradients over the LV volume at all time frames.

**Figure 2:**
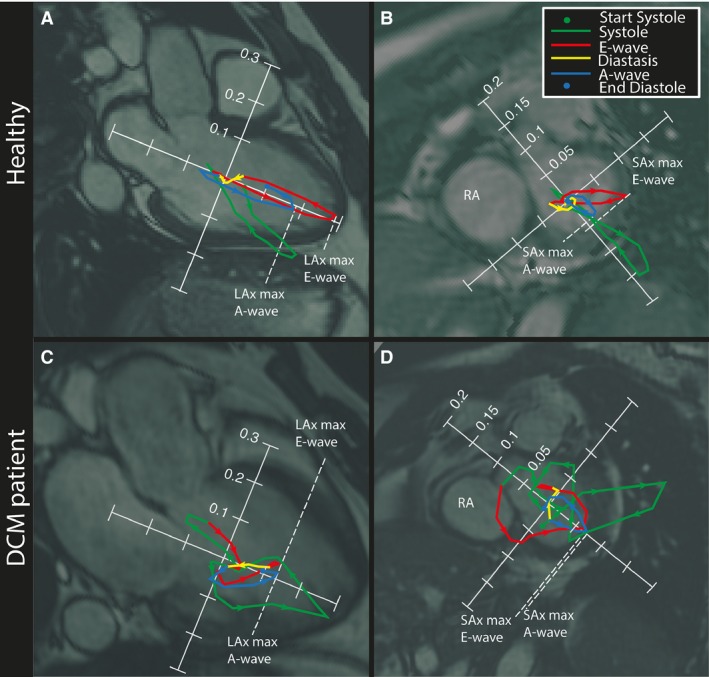
Hemodynamic forces [N] over the cardiac cycle, projected onto long‐axis (LAx) (A and C) and short‐axis (SAx) images (B and D). The cardiac cycle is color‐coded as follows: systole (green), early diastole (E‐wave, red), diastasis (yellow), and late diastole (A‐wave, blue). Arrows along the force curve indicate the direction over time. Panels A and B show a healthy 54‐year‐old male; panels C and D) show a 43‐year‐old male with dilated cardiomyopathy. The maximum values for E‐ and A‐wave indicated by the dashed white lines in the LAx‐direction (A and C) and in the SAx‐direction (B and D) were used to calculate the “SAx‐max/LAx‐max force”‐ratio. The ratio was calculated by dividing the maximum force along the apex‐to‐base axis in the LAx plane with the maximum force along the anteroseptal‐to‐inferolateral axis in the SAx‐plane. LA, Left Atrium; LV, Left Ventricle; RA, Right Atrium.

At all time frames of the cardiac cycle the LV was semiautomatically segmented from the morphological SAx‐images guided by LAx‐images (Segment, Medviso AB, Lund, Sweden). In this study, the LV volume was defined from the SAx‐images where the endocardial contour of the compact ventricular myocardium was outlined. In the most basal slice, as much of the LV volume as possible was included without including any LA volume. The LVOT was not included in the LV volume (Fig. 1). The segmentation was used to create a binary mask that was resampled to fit the resolution of the velocity data. The mask was temporally resampled by the use of nearest neighbor interpolation. The segmentation and the 4D flow data were used to calculate the pressure gradients from the Navier‐Stokes equation (the body forces, f, were excluded): $\nabla p=-\rho \frac{\partial v}{\partial t}-\rho v\nabla v+\mu \nabla^{2}v$, where *v* [m/s] is the velocity, *ρ* the density (1060 [kg/m^3^]) and *μ* the viscosity (0.004 [Ns/m^2^]) of blood. The blood is assumed to be a Newtonian and uncompressible fluid. By integrating the pressure gradients, ∇p, over the entire LV volume at every time frame, the 3D hemodynamic force vector was calculated for every time frame of the cardiac cycle (Fig. 1). The force was projected onto a plane corresponding to a standard LAx three‐chamber plane (Fig. 2) and a basal SAx‐image. The reference point was set to the center of mass of the LV (projected on the three‐chamber plane and the SAx‐plane) at a time in early diastole (Fig. 2).

**Figure 3:**
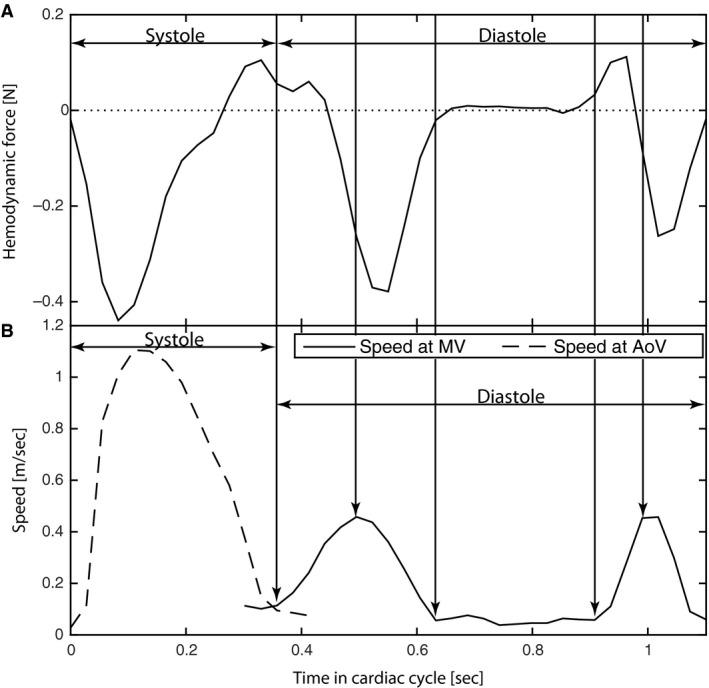
(A) The magnitude of the hemodynamic force [N] with apex direction as negative and LA‐direction as positive. (B) Speed over time, where the velocities are extracted from the 4D flow dataset at a point in the mitral (MV) valve inflow (solid black line) and a point in the aortic valve (AoV) outflow (dashed black line). The different phases of the cardiac cycle are defined from the velocities at these locations. The data are from a healthy 42‐year‐old male. The vertical black arrows indicate, from left to right, end of systole, peak E‐wave, onset of diastasis, end of diastasis, and peak A‐wave.

Speed data were extracted from two points set near the centers of the mitral valve (MV) orifice and the LV outflow tract at end diastole. The plot of the speed data over time was used to do a rough division of the cardiac cycle into subphases and to describe the timing of the cardiac cycle (Fig. 3); this was confirmed with visualizations of transvalvular flow in the three‐chamber orientation made with streamlines emitted for 25 ms. The cardiac cycle was divided into systole and diastole with subphases (E‐wave, diastasis and A‐wave) (Fig. 3). E‐wave was defined as the time between the onset of the increase in speed in the MV plot during diastole to the point where the decrease in speed ends, confirmed visually by blood flow through the mitral valve. Diastasis was defined as the time between the points where E‐wave ended and A‐wave started. A‐wave was defined as the time between the onset of speed increase following diastasis to the point where the decrease in speed ends, and confirmed visually by the second phase of blood flow through the mitral valve.

In order to assess the distribution of hemodynamic force directions, the “SAx‐max/LAx‐max” force ratio was calculated at both early (E‐wave) and the late (A‐wave) diastolic filling. The ratio was calculated by dividing the maximum force along the apex‐to‐base axis in the LAx plane (i.e., the force vector projected on to the apex‐to‐base axis) with the maximum force along the anteroseptal‐to‐inferolateral axis in the SAx‐plane (i.e., the force vector projected on to the anteroseptal‐to‐inferolateral axis) (Fig. 2). The maximum values were extracted from the time frames corresponding to early (E‐wave) and late (A‐wave) diastolic filling.

To further assess the direction of the global LV hemodynamic forces with respect to the main flow axes, the LV hemodynamic force perpendicular to the apex‐to‐base axis was assessed over the whole diastolic phase. Therefore, for each subject, the time‐averaged magnitude of the force in this direction was computed by integrating the magnitude of the force in the SAx‐plane over (1) the early filling phase and (2) the late filling phase, which was divided by the number of time frames.

### Statistical comparisons

Data are presented as mean ± 1 standard deviation (SD), unless otherwise stated. Intergroup comparisons were made for the (1) clinical parameters; (2) the “SAx‐max/LAx‐max‐force”‐ratio at E‐ and A‐wave; and (3) the mean SAx‐forces, by the use of group‐wise comparisons with unpaired t‐tests. Linear regression was used to test the relation between the “SAx‐max/LAx‐max” force ratio and sphericity index (SI). A *P*‐value <0.05 was considered significant. All statistical calculations were performed in Matlab (R2014b, The MathWorks Inc., Natick, MA).

## Results

There was no difference in age and heart rate, whereas parameters of LV characteristics such as ejection fraction, SI and end‐diastolic volume (EDV) differed between the two groups (Table 1). Based on echocardiographic criteria, all 10 healthy subjects and five of 10 patients showed normal LV diastolic function, two, the presence of diastolic dysfunction was based on echocardiography criteria patients had LV relaxation abnormality, one had pseudonormal filling, and two had restrictive filling at rest. No patient had more than mild mitral regurgitation.

### Hemodynamic forces in the healthy LV

**Figure 4:**
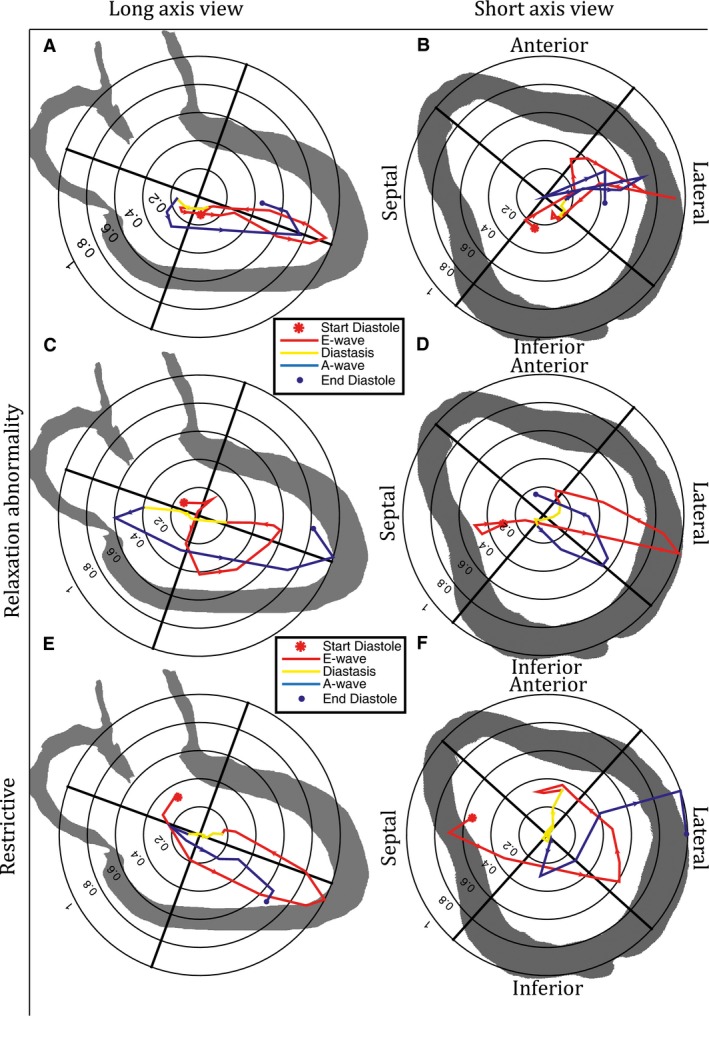
Hemodynamic forces projected on a three‐chamber (A, C, and E) plane and a SAx‐plane (B, D and F). Panels A and B: The magnitude of the force was normalized by the maximum force during the diastolic phase for each of the ten healthy subjects. The data were then resampled to the same number of data points for each subject and the mean magnitude and direction were calculated. Panels C and D: A 62‐year‐old. male DCM patient with LV diastolic relaxation abnormality. Panels E and F: A 61‐year‐old. female DCM patient with LV restrictive diastolic filling. The individual patient data were normalized by the maximum force in each direction. Arrows along the curve indicate the temporal direction of the force plot over diastole, from onset diastole (red *) to end diastole (blue ●).

**Figure 5:**
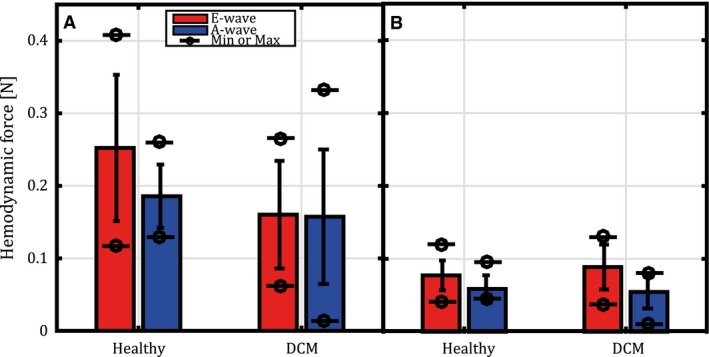
The mean (columns), standard deviation (error bars) and range (minimum and maximum values indicated with circle/line marker) of the maximum LV hemodynamic forces in healthy subjects (*n* = 10) and DCM patients (*n* = 10). Red color indicates early diastolic filling (E‐wave) and blue color indicates late diastolic filling (A‐wave). Maximum force in (A) LAx‐plane and (B) SAx‐plane.

In the healthy subjects, the hemodynamic forces projected on to the three‐chamber LAx plane, presented a consistent ellipsoid‐like pattern (Fig. 4A and Fig. S1); at onset of diastole and throughout the first part of early filling, the force increased along the base‐to‐apex axis, first in the base direction and then turning in the apical direction with an increase in magnitude (Figs. 3 and 4A). Among the subjects, the directional pattern was consistent during early filling (supplemental material), but there was a wider range in the magnitude of the hemodynamic force (Fig. 5A). After the force peak, the force diminished as the LV pressure equilibrated with the onset of diastasis (Figs. 3 and 4A). The peak velocity of early filling as defined from registration at the mitral valve preceded the peak in the force curve by approximately 25–50 ms. In 8 of 10 healthy subjects the early filling force curve moved in a counter clockwise direction from the LAx‐perspective.

In the SAx‐view (Fig. 4B and Fig. S2), the pattern of the forces was more asymmetrical and less consistent with respect to direction of the peak force compared to the pattern in the LAx‐view. For the SAx‐view there was also a wide range in force magnitude between the different subjects (Fig. 5B). During diastasis, the force was around zero with no apparent pattern regarding direction.

At onset of late diastolic filling, a force directed toward the atrium (Figs. 3, 4A and Fig. S1) was observed in the LAx‐view in the majority of the healthy subjects. In all 10 subjects, the force curve moved in a counter clockwise direction during late filling, from atrium down toward the apex along the apex‐to‐base axis. The magnitude of the force differed between the subjects but the range was smaller during late compared to early filling (Fig. 5A) as well as the observed maximum force in the LAx‐view (Figs. 4A and 5A).

Similar to the characteristics during early filling, the forces during late filling in the SAx‐view showed a less consistent pattern in terms of direction compared to the pattern in the LAx‐view (Fig. 4B and Fig. S2). The range of the magnitude in the SAx‐view was slightly greater for early compared to late filling (Fig. 5B). In four of the subjects the time of the force peak and the velocity peak coincided as compared to none of the subjects during early filling. In all subjects the base‐to‐apex direction was the main directional axis of the hemodynamic force throughout diastole.

At the onset of systole, the magnitude of the hemodynamic force is small. During the upslope of systole it increases and is oriented opposite to the outflow direction. During late systole the force decreases, while maintaining the same direction. At end‐systole, a slight shift toward the direction of outflow is observed (Figs. 2 and 3).

### Hemodynamic forces in the Healthy versus the DCM group

**Figure 6:**
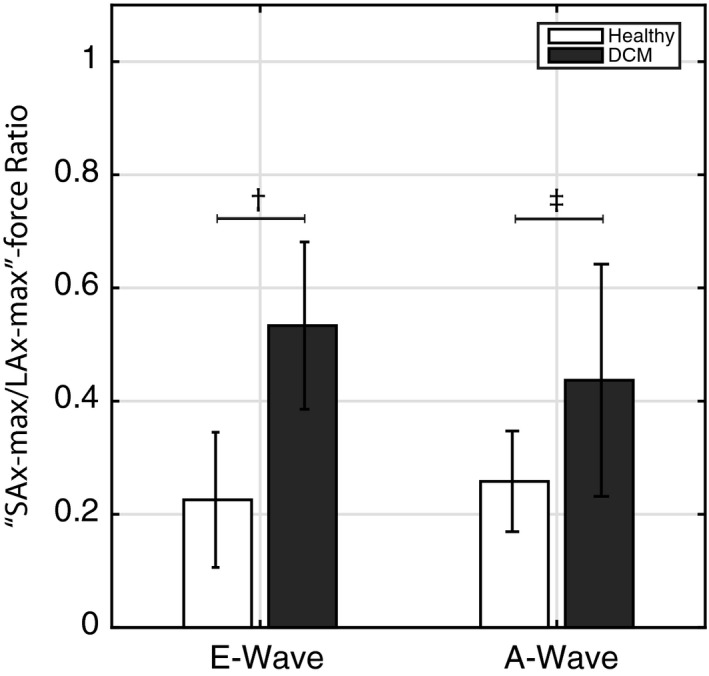
The “SAx/LAx‐max force”‐ratio is larger at both E‐ and A‐wave for the DCM patients (black bars) compared to the healthy subjects (white bars). ^†^ *P* < 0.0001 and ^‡^ *P* < 0.03. SAx, short axis; LAx, long axis.

The patient group was heterogeneous with respect to LV characteristics such as of the severity of diastolic and systolic dysfunction and remodeling. Overall, the trajectories of the forces present a more heterogeneous pattern compared to the healthy subjects (Figs. 2 and 4, Supplemental material). The range of the magnitude appeared smaller at early filling compared to late filling in the LAx‐view in the DCM group (Fig. 5A). At late filling, the range was larger compared to the healthy subjects, particularly from the LAx‐perspective (Fig. 5A and B). The “SAx‐max/LAx‐max”‐ratio was computed in order to assess the distribution of the hemodynamic force directions. This ratio (Fig. 6) was significantly larger (implying less base‐to‐apex orientation) at both early diastolic filling (0.53 ± 0.15 vs 0.23 ± 0.12, *P* < 0.0001) and late diastolic filling (0.44 ± 0.21 vs. 0.26 ± 0.09, *P* < 0.03) in the patient group compared to healthy subjects (Fig. 2).

The time‐averaged magnitude of the force in the SAx‐plane was significantly higher in the patient group during the early filling phase (0.045 ± 0.011 vs. 0.032 ± 0.007, *P* = 0.011). No intergroup difference was observed during the late filling phase (0.032 ± 0.014 vs. 0.034 ± 0.012, *P* = 0.76).

There was a weak to moderate relation between SAx‐max/LAx‐max force ratio and SI at early (*R* ^2^ = 0.214, *P* = 0.04) and late (*R* ^2^ = 0.343, *P* = 0.007) filling using linear regression analysis.

The “SAx‐max/LAx‐max” force ratio during early filling was higher in the subgroup of patients with normal diastolic function (*n* = 5) compared to the healthy subjects (0.58 ± 0.11 vs. 0.23 ± 0.12, *P* < 0.0001), whereas no difference was observed during late filling (0.26 ± 0.09 vs. 0.37 ± 0.1479, *P*‐value = 0.0903).

## Discussion

In this study, the hemodynamic filling force of the LV blood acting on the myocardial wall was calculated in a group of healthy subjects and compared to a group of clinically compensated idiopathic dilated cardiomyopathy patients with different stages of LV dysfunction. The ratio of forces acting in the LAx‐ and SAx‐directions was altered in the DCM group at early and late diastolic filling, indicating that the hemodynamic forces act to a greater extent orthogonally to the main flow directions compared to normals.

Hemodynamic forces computed from 4D flow MRI data allow for comprehensive assessment of LV blood flow dynamics over the cardiac cycle. In the healthy subjects the hemodynamic forces showed a consistent pattern in terms of direction, while the range in magnitude was wide. This range could be explained by several factors such as variable LV size, heart rate, and age‐dependent LV myocardial relaxation.

During LV diastolic filling, blood enters the LV, where the myocardium will exert a decelerating force on the blood. The blood will exert an opposite force on the myocardium directed toward the apex concordant with the main flow direction during filling. During LV systole, the myocardium will contract and accelerate the blood toward the LV outflow tract; this blood will in turn exert a force on the myocardium in the direction opposite the outflow direction. Accordingly, the resistance (i.e., the afterload) the LV has to overcome will be represented as a force opposing the outflow direction. Blood that has left the LV cavity will exert forces on the walls of the aortic root and the proximal ascending aorta, but this is not assessed in this study as only the data within the LV as defined in the methods section is analyzed. Consequently, the LV hemodynamic force, which is the focus of this study, will be dominated by the force due to acceleration of the blood during systole and deceleration of blood during diastole (Fig. 3). Moreover, as the LV hemodynamic force is related to blood flow, the changes in the absolute pressure during isovolumic contraction will not be reflected in the hemodynamic force. The regional hemodynamic forces due to this pressure rise are directed perpendicular to the LV wall. Therefore, these forces will cancel out when assessed from the global LV perspective.

During early filling the force of the LV intracavitary blood was observed to move counter clockwise from the perspective of the LAx‐view in 8 of the 10 healthy subjects. This finding is consistent with previous studies that have shown that a substantial portion of the early inflow enters the LV along the inferolateral wall before turning toward the anterior septum (Bolger et al. 2007; Eriksson et al. 2011). The temporal displacement between the inflow velocity peak and the force peak at early filling may be caused by the fact that the velocity peak is measured at the MV orifice, whereas the hemodynamic forces reflect the flow dynamics in the whole LV.

Interestingly, at the beginning of late filling the force of the LV blood is directed toward the LA, compared to a smaller atrially directed force at the beginning of the early filling phase. This difference may reflect the distinct filling mechanisms between the two diastolic filling phases. As the atrial contraction accelerates blood residing in the atrium, the blood in the LV will exert a force opposed the accelerating inflowing blood. However, the blood residing in the LA is only accounted for in the calculation of the LV hemodynamic force once it has entered the LV. As a consequence the opposing force seen at the first few time frames in late diastole is later “overrun” when a substantial portion of the late inflowing blood is directed toward the apex along the base‐to‐apex direction. Another contributing factor might be that blood that entered during early filling has turned in the apical or midventricular region of the LV cavity and is directed up toward the basal region and that the force of this blood portion exceeds the force of the blood of the early phase of late inflow. As the late inflow proceeds the acceleration toward the apex will dominate and the net force of the LV blood will be directed toward the apex. The differences in filling mechanisms were also seen in the MV‐to‐apex pressure in normal subjects (Eriksson et al. 2014).

While hemodynamic forces in normal LVs are directed concordant with the main flow directions, the findings from the SAx/LAx force ratios in the myopathic LVs propose that the forces are orthogonal to the main flow direction to a greater extent especially during early, but also during late filling. One may expect that this is less efficient from a LV pumping point of view and may exert a larger force on the myocardial wall. As opposed to the configuration of normal LVs, the remodeled LVs of the DCM patients present a more spherical shape. These geometrical differences, in combination with different LV filling characteristics, may contribute to abnormal diastolic flow within LVs of DCM patients (Hill and Olson 2008; Carlhäll and Bolger 2010). One example is a more prominent central vortex with a circular motion that is evident throughout diastole in DCM LVs (Mohiaddin 1995). Moreover, a remodeled LV can affect the geometry of the mitral subvalvular apparatus and thus LV inflow characteristics. An alteration in LV inflow direction at early filling that influences intraventricular flow patterns may contribute to the higher mean force in the SAx‐plane in the patients during early filling. No difference in the mean force in the SAx‐plane was demonstrated during the late filling phase, possibly due to the large variability in late diastolic conditions among the patients. It is reasonable to expect that intergroup differences in forces in the SAx‐plane will not be seen over the entire diastolic phase, as, for example, during diastasis there are very small forces exerted by the blood.

Given that both the “SAx‐max/LAx‐max” force ratio and the SI are higher in the DCM compared to healthy subjects, it is reasonable to expect some relation between these two variables. Linear regression analysis for early and late filling showed only a weak to moderate relation between them, however, suggesting that there are additional factors to SI explaining the force ratio.

It appears that different diastolic dysfunction related filling patterns are reflected by the magnitude of the hemodynamic force at the respective filling phases (early and late filling). For instance, an elevated early/late velocity magnitude ratio due to a relaxation abnormality is also reflected by an elevated early/late force magnitude ratio. However, the direction of the forces appears to be less related to conventional indices of diastolic dysfunction. Interestingly, the force ratio at early filling was significantly higher in patients with normal LV filling patterns compared to healthy subjects, whereas no difference was observed during late filling. Alterations in LV inflow direction during the early filling phase that influence intracavitary flow patterns may contribute to the increased force ratio. The number of patients with different degrees of abnormal LV filling patterns defined by echo Doppler was very low and did not allow evaluation of any relationship between specific levels of diastolic dysfunction and changes in hemodynamic forces. Of note, we do not expect any correlation of specific LV filling pattern with the underlying etiology of DCM in the current patient cohort.

Given that LV force is a volume integral, it is interesting to observe that while the end‐diastolic volume is significantly larger in patients compared to healthy subjects (Table 1), there is a trend that the maximum force values for E‐ and A‐wave are smaller in patients compared to healthy (Fig. 5).

Assessment of LV hemodynamic force may add to pathophysiological understanding of progressive global remodeling of the failing heart. The interaction between the flowing blood and the developing heart chambers stimulates a continuous positive remodeling process that creates an optimal geometry for efficient flow (Kilner et al. 2000; Hove et al. 2003; Richter and Edelman 2006; Yashiro et al. 2007). However, the same responses to flow‐induced forces that shape the developing heart can also play a role in the development and progression of the adverse cardiac remodeling in postnatal life which is a key component of heart failure (Hove et al. 2003; Thum et al. 2007; Davies 2009). We speculate that altered hemodynamic forces during diastole could be a contributing factor to elevated diastolic wall stress, which in turn is a determinant of the development of adverse cardiac remodeling (Mann 2004). The forces in the healthy subjects had an overall higher magnitude, and the pattern in terms of direction was rather consistent, compared to the forces in DCM patients. We believe that the heterogeneity of the direction of the force is more important than the absolute force values as a contributor to the development of pathological remodeling. An altered interaction between the LV hemodynamic force and myocardium may be a part of a “vicious circle” where a chamber with some global adverse remodeling/increased sphericity shows a larger “SAx‐max/LAx‐max” force ratio, with relatively higher global force that is disconcordant with the predominant LV flow direction; this may in turn contribute to progression of the adverse remodeling and sphericity.

The utility of the assessment of LV hemodynamic forces in cardiac disease will be evaluated in future studies. Potential areas of application may be to assess restoration of normal homogenous LV hemodynamic force patterns in remodeled and dysfunctional LVs using optimized pacing and cardiac resynchronization (Pedrizzetti et al. 2015), and also to refine design and optimize orientation of MV prostheses to preserve normal homogenous LV hemodynamic force patterns. Moreover, assessment of the magnitude and heterogeneity of LV hemodynamic force across a range of heart rates in normal and myopathic ventricles may provide new perspectives on the optimization of target heart rate in heart failure patients, if future work can establish that these alterations in hemodynamic force contribute to progressive adverse LV remodeling.

### Limitations

The study population of 20 subjects is relatively small. The subset of healthy subjects showed consistent force patterns, however, suggesting that this is an adequate comparator to the patient group. DCM patients can be expected to be heterogeneous with respect to many relevant criteria, including ventricular size, myocardial function and load, and this small number of patients is rather representative of that clinically relevant spectrum.

Acquisition time of 4D flow MRI can be long. The scan with longest duration in this study was 57 min and was caused by ECG problems. However, by the use of new scanner hardware and recent 4D flow sequences, scan times may be significantly reduced with preserved data quality (Petersson et al. 2016).

Segmentation was performed on short‐axis images reconstructed in 30 time frames, whereas the 4D flow MRI were reconstructed to 40 time frames to ensure that all temporal information present is the acquisition is preserved. The segmented masks have therefore been resampled to 40 time frames using a nearest neighbor interpolation. Preliminary tests show that the contribution of the border voxels to hemodynamic force is small, and therefore we do not expect any significant changes to the results due to this interpolation. However, for future studies, we recommend using the same number of reconstructed time frames for both acquisitions.

The user‐dependent steps of the presented method are: 1) the segmentation of the LV; previous 4D flow MRI studies including segmentation of the LV blood volume have shown small inter and intraobserver variability of subvolumes of LV flow (Eriksson et al. 2010); and 2) setting the timing reference points in the LV inflow and outflow regions.

The temporal resolution of the velocity data was 50.4 ms. While better temporal resolution would be preferable, particularly in patients with higher heart rates, this level represents an acceptable tradeoff between acquisition time and temporal resolution.

The viscous term in Navier–Stokes equations is underestimated, it is most important when assessing parameters in the shear layer close to the walls, and should be a minor determinant of the forces examined in this study.

Electrocardiography and echocardiography are not as sensitive as other methods such as CMR in the detection of small areas of ischemia/infarction. However, it is reasonable to believe that small areas of ischemia/infarction would not have a significant impact on the global hemodynamic force patterns. It is likely that larger areas of ischemia/infarction would be detectable as regional wall motion abnormalities using echocardiography, and might impact the hemodynamic forces. Patients with regional wall motion abnormalities were excluded from this series.

Although these novel and fundamental aspects of cardiac flow physiology do not provide mechanistic insights as to whether the abnormal LV hemodynamic forces in DCM are contributors to or consequences of adverse remodeling, this study facilitates further mechanistic/translational studies including evaluation of LV hemodynamic forces in clinically important settings with altered LV Inflow directions (modulation of valve apparatus) or LV wall motion patterns (pacing). Insight regarding the clinical potential of this new method may be obtained by relating the degree of force abnormality with other accepted measures of cardiac performance and decompensation, such as 6‐minute walk test or maximal oxygen consumption, awaits further study.

## Conclusions

This study demonstrates that 4D flow MRI data allow quantification of LV hemodynamic forces acting on the myocardium. The LV hemodynamic filling forces showed a similar pattern across healthy subjects, with the main hemodynamic force directed along the base‐to‐apex axis, whereas in the DCM group the distribution in direction was heterogeneous during early and late diastole. Assessment of flow‐based forces may be a marker of altered hemodynamic effects on the myocardial wall, and thus, a novel tool in the study of progressive adverse cardiac remodeling.

## Conflict of Interest

The authors declare that they have no conflict of interest.

## Supporting information




**Figure S1.** Polar plots of the hemodynamic force over time projected on a plane corresponding to a standard long‐axis three‐chamber image. Ten healthy subjects (N01–N10) and 10 dilated cardiomyopathy patients (P01–P10) are included. The forces are presented in N and the cardiac cycle is colored according to: systole, green; early diastole (E‐wave), red; diastasis, yellow; and late diastole (A‐wave), blue.

**Figure S2.** Polar plots of the hemodynamic force over time projected on a plane corresponding to a standard basal short‐axis image. Ten healthy subjects (N01–N10) and 10 dilated cardiomyopathy patients (P01–P10) are included. The forces are presented in N and the cardiac cycle is colored according to: systole, green; early diastole (E‐wave), red; diastasis, yellow; and late diastole (A‐wave), blue.
