# Supplementary figures and images for: Assessment of left ventricular hemodynamic forces in healthy subjects and patients with dilated cardiomyopathy using 4D flow MRI
Source: Physiol Rep. 2016 Feb 1;4(3):e12685. doi: 10.14814/phy2.12685 (PMC4758930; doi:10.14814/phy2.12685)

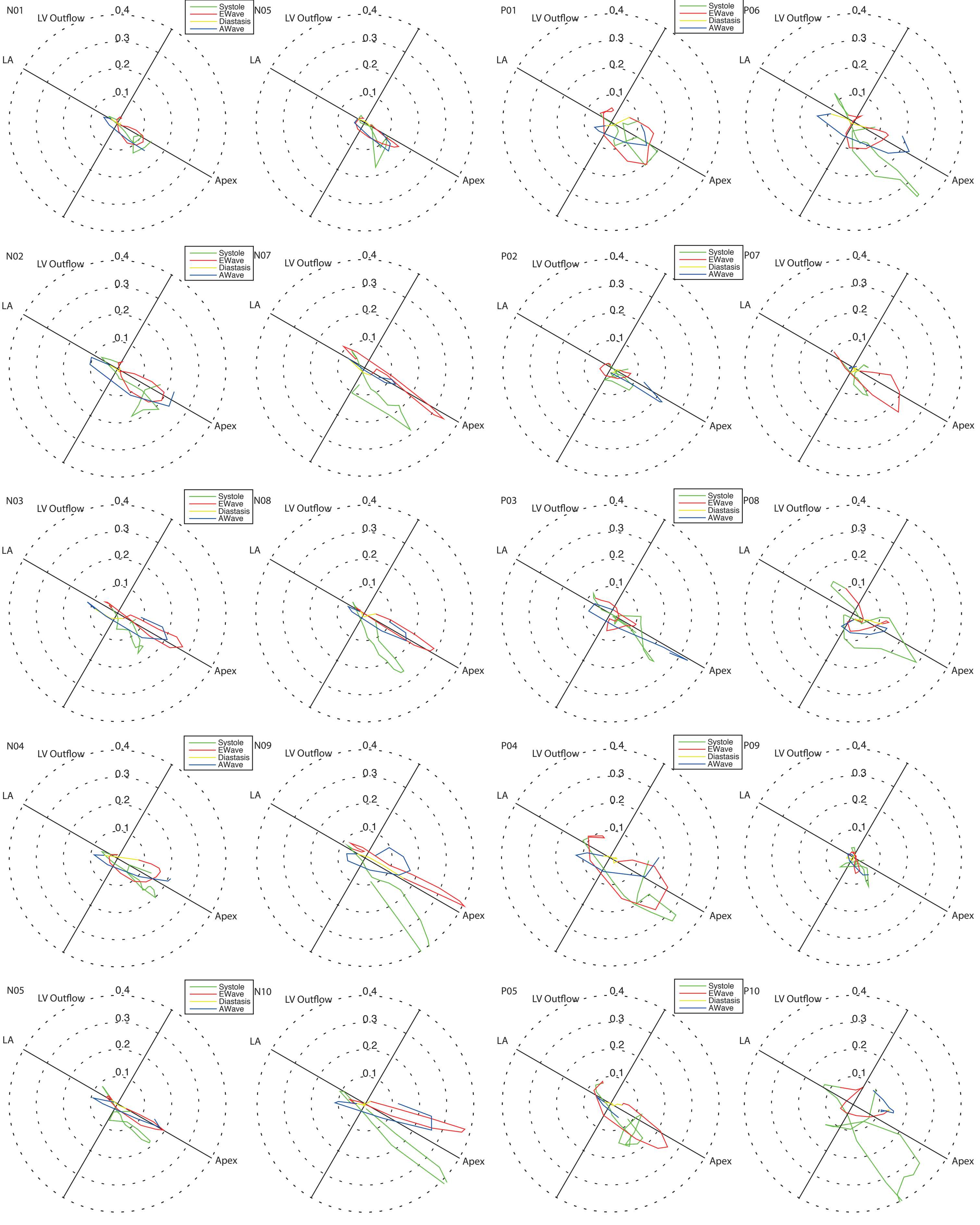

Supplement: Supplementary file 1 — Figure S1. Polar plots of the hemodynamic force over time projected on a plane corresponding to a standard long‐axis three‐chamber image. Ten healthy subjects (N01–N10) and 10 dilated cardiomyopathy patients (P01–P10) are included. The forces are presented in N and the cardiac cycle is colored according to: systole, green; early diastole (E‐wave), red; diastasis, yellow; and late diastole (A‐wave), blue. [file PHY2-4-e12685-s001.pdf]

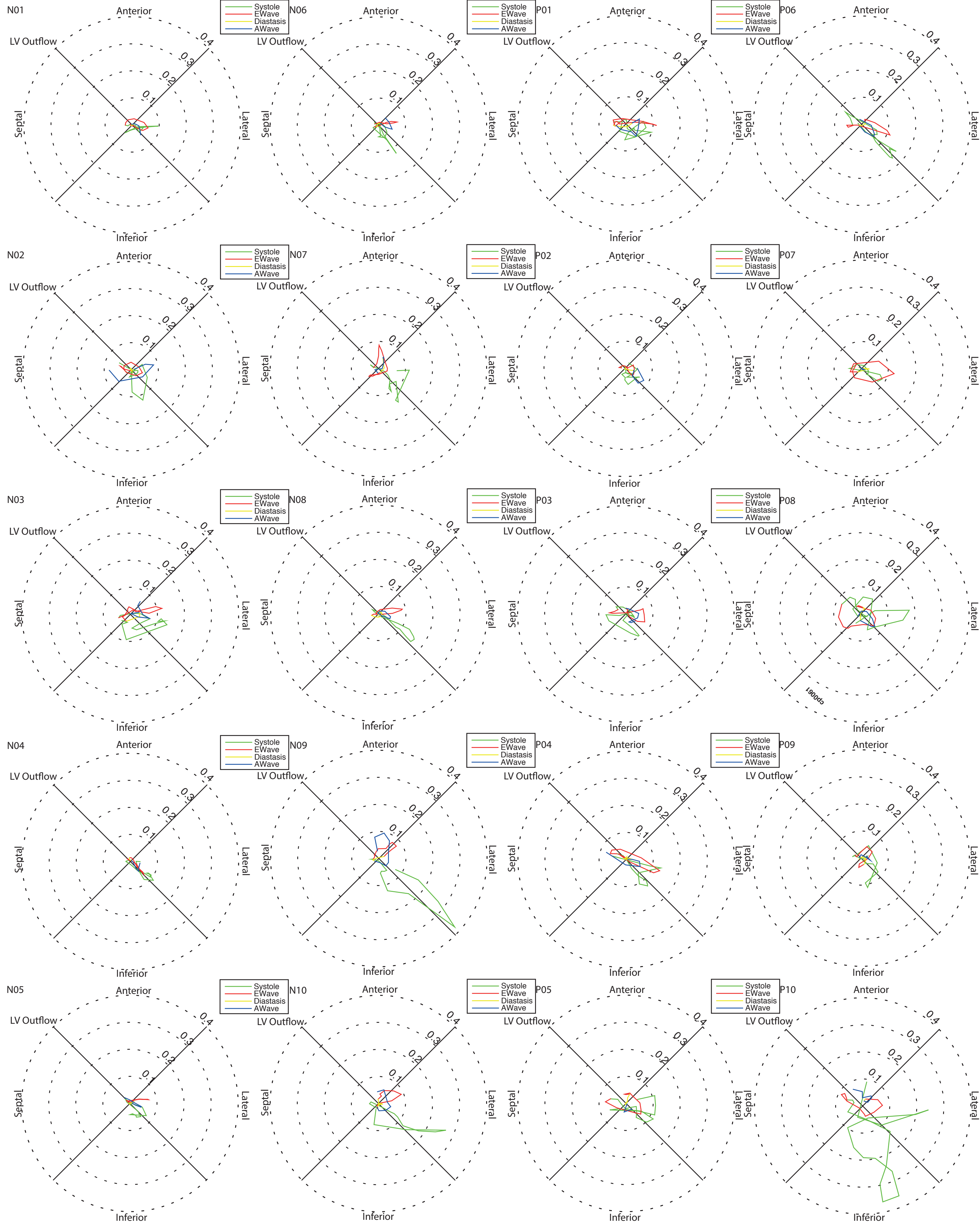

Supplement: Supplementary file 2 — Figure S2. Polar plots of the hemodynamic force over time projected on a plane corresponding to a standard basal short‐axis image. Ten healthy subjects (N01–N10) and 10 dilated cardiomyopathy patients (P01–P10) are included. The forces are presented in N and the cardiac cycle is colored according to: systole, green; early diastole (E‐wave), red; diastasis, yellow; and late diastole (A‐wave), blue. [file PHY2-4-e12685-s002.pdf]
